# Family resilience and demoralization in decompensated cirrhosis: parallel mediation of psychological resilience and social support

**DOI:** 10.3389/fpsyg.2025.1623122

**Published:** 2025-08-01

**Authors:** Haixia Gao, Gang Mao, Xuelian Gu, Hui Wang, Jianhua Niu, Lei Liu

**Affiliations:** ^1^School of Nursing, Shandong First Medical University & Shandong Academy of Medical Sciences, Jinan, China; ^2^Department of Critical Care Medicine, The Fourth People’s Hospital of Jinan, Jinan, China; ^3^Medical Insurance Office, The Third People’s Hospital of Jinan, Jinan, China; ^4^Department of Rheumatology and Immunology, The Fourth People’s Hospital of Jinan, Jinan, China; ^5^Department of Hematology, The Fourth People’s Hospital of Jinan, Jinan, China; ^6^Department of Gastroenterology, The Fourth People’s Hospital of Jinan, Jinan, China

**Keywords:** decompensated cirrhotic patients, demoralization syndrome, family resilience, psychological resilience, social support, parallel mediation effect

## Abstract

This study aims to understand the relationship between psychological resilience and social support in family resilience and demoralization syndrome (DS) in decompensated cirrhotic patients and verify whether there is a parallel mediation effect. A cross-sectional design was adopted, and a questionnaire was administered to 260 patients with decompensated cirrhosis in Jinan, Shandong Province, China. The Mandarin Version of the Demoralization Scale (DS-MV) was used to assess DS. Spearman’s correlation was used to analyze the relationship between family resilience, DS, psychological resilience, and social support, and predictors of DS were explored using multiple linear regression. Parallel mediation effect analysis was performed using a bootstrap test. The proportion of patients with severe DS was 18.46% of those with decompensated cirrhosis. Spearman correlation analysis demonstrated that DS was negatively correlated with psychological resilience, family resilience, and social support (*r* = −0.738, *p* < 0.01; *r* = −0.668, *p* < 0.01; *r* = −0.405, *p* < 0.01). Multiple linear regression analysis showed that psychological resilience (*β* = −0.477, *p* < 0.001), family resilience (*β* = −0.364, *p* < 0.001), and social support (*β* = −0.108, *p* = 0.01) could influence DS of decompensated cirrhotic patients. Bootstrap analysis confirmed a significant parallel mediation effect of psychological resilience and social support (95% CI: −0.999 to −0.499), accounting for 51.93% of the total effect. Family resilience had a significant direct effect (95% CI: −0.995 to −0.356), accounting for 48.07% of the total effect. Therefore, attention should be paid to DS in decompensated cirrhotic patients. Family resilience, psychological resilience, and social support can be used to decrease the level of DS.

## 1 Introduction

Liver cirrhosis is an irreversible and chronic progressive hepatic disorder; it is an important cause of morbidity and mortality in patients with chronic liver disease (Huang et al., 2023b). It was associated with 2.4% of global deaths in 2019 (Huang et al., 2023b). Liver cirrhosis is a prevalent and increasing public health challenge worldwide (Huang et al., 2023a). Patients with compensated cirrhosis progress to decompensated cirrhosis at a rate of 5 to 7% per year (Sun et al., 2018). Patients with decompensated cirrhosis have a 1-year probability of proceeding directly to death of 20% (Fleming et al., 2010).

The concept of “demoralization” was first introduced by the renowned American psychologist Frank JD in 1974 and was characterized by feelings of impotence, isolation, and despair stemming from persistent failure to cope with internally or externally induced stresses the person and those close to him expect him to handle (Frank, 1974). In the early 21st century, Clarke and Kissane formally proposed the concept of demoralization syndrome (DS). DS is a negative psychological state observed in patients and is primarily characterized by manifestations of helplessness, hopelessness, existential meaninglessness, and perceived incompetence (Clarke and Kissane, 2002; Kissane et al., 2001). Studies have demonstrated that DS significantly impairs patients’ emotional regulation and diminishes their capacity to cope with adverse life events, leading to a marked reduction in health-related quality of life (Nanni et al., 2018). In severe cases, this syndrome is associated with an elevated risk of suicidal ideation and behaviors (Zhu et al., 2022). Current DS research has primarily focused on patients with cancer and end-stage patients with other chronic diseases (Robinson et al., 2015). Decompensated cirrhotic patients frequently develop complications including gastrointestinal bleeding, infections, and ascites. They also face an elevated cancer risk, collectively impairing quality of life (Nagel et al., 2020). Marked by a protracted course, complex symptomatology, and recurrent exacerbations, this condition predisposes patients to anxiety, negative treatment perceptions, and suicidal ideation or behavior (Ou et al., 2022). Significant social stigma (Wahlin and Andersson, 2021) often culminates in exclusion or isolation (Schomerus et al., 2022). Negative emotions and social isolation in decompensated cirrhotic patients may predispose them to the development of DS. Despite the extensive research on DS in cancer populations, its mechanisms in decompensated cirrhosis remain underexplored. Therefore, investigating the mechanisms underlying DS in patients with liver cirrhosis has become critical.

Family resilience, also known as family adaptability or resilience, refers to the family’s positive coping ability in the face of major adversity or crisis (Silverman and Grunauer, 2020). Studies have shown that strong resilience enables families and individuals to adapt to illness effectively (Wang Y. et al., 2024), fostering stability in family functioning and facilitating patient recovery.

Perceived social support refers to an individual’s subjective perception and evaluation of the degree of support received from the external world, which differs from actual social support (Zimet et al., 1990). Previous studies have shown that family resilience is positively related to perceived social support and that family resilience can help patients make better use of social support systems (Chen et al., 2021).

Psychological resilience is an adaptive process individuals demonstrate when confronting adversity, trauma, tragedy, threats, or significant stressors (American Psychological Association, 2014). It can help patients manage stressful situations and improve their quality of life (García-Martínez et al., 2021). A study discovered that unstable family environments could undermine psychological resilience in family members (Al-Smadi et al., 2024). Research indicated that for parents of children with acute lymphoblastic leukemia, interventions strengthening family functioning and resilience are crucial to supporting parental psychological well-being during treatment (Ferraz et al., 2025). Wang Y. et al. (2024) discovered that family resilience significantly influences social support and psychological resilience among patients undergoing maintenance hemodialysis. Research indicates that patients with ovarian cancer with higher levels of psychological resilience and social support during targeted therapy experience correspondingly lower levels of DS (Tao et al., 2025).

Currently, no research data are available on the correlation between family resilience and DS in decompensated cirrhotic patients, and previous studies have found a negative correlation between the two (Yan et al., 2023). Thus, this study (1) verifies the relationship between family resilience, psychological resilience, social support, and DS in patients with decompensated cirrhosis, (2) explores whether psychological resilience and social support play parallel mediating roles between family resilience and DS, and (3) provides novel insights and a practical basis for developing psychological interventions to improve the mental health of patients with decompensated cirrhosis. Therefore, we propose the following hypothesis:

*Hypothesis*: Psychological resilience and social support play parallel mediating roles in the relationship between family resilience and DS.

## 2 Materials and methods

### 2.1 Study design and sampling

This cross-sectional study adopted a convenience sampling method (Shi et al., 2023). A paper-based questionnaire survey was conducted with decompensated patients with cirrhosis in a Class A tertiary comprehensive hospital in Jinan. The data were collected between August 2024 and March 2025. The inclusion criteria were as follows: (1) the pathological diagnosis was decompensated cirrhosis; (2) age≥18 years; (3) clear consciousness; (4) informed consent indicating willingness to participate in the study. The exclusion criteria were as follows: (1) diagnosis of primary hepatocellular carcinoma through clinical histopathological examination; (2) clinical instability or rapid deterioration; (3) diagnosis of a mental disorder or language communication disorder; (4) major life events within the past 3 months. The required sample size was calculated using PASS software (version 15.0). Since multiple regression analysis was used to explore influencing factors, the corresponding effect size method in PASS was applied. The results indicated that with an effect size of 0.15, a power of 90%, and a statistical significance level of 5%, the minimum required sample size was 236 participants. No consensus exists regarding structural equation modeling sample sizes, though literature standards suggest 200–500 as generally appropriate (Wu, 2016).

### 2.2 Data collection procedure

Patients with decompensated cirrhosis were recruited at public hospitals using convenience sampling between August 2024 and March 2025. Participants signed an informed consent form and completed the questionnaire. It took approximately 20–30 min to complete the paper questionnaire. For patients who could not complete the questionnaire independently, the investigator would provide technical support for questionnaire comprehension. Investigators were strictly trained to avoid influencing responses. The investigators assisted patients primarily to ensure the accessibility of questionnaire completion, especially for those with low literacy or visual impairment. Family members were not allowed to assist in this aspect.

### 2.3 Measurements

#### 2.3.1 Demoralization syndrome

The Demoralization Scale-Mandarin version (DS-MV) was used to measure the level of DS in patients and was translated from the demoralization scale developed by Kissane et al. (2004). The DS-MV comprises 24 items across five subscales: loss of meaning, dysphoria, disheartenment, helplessness, and sense of failure. Each item is rated on a 5-point scale, ranging from 0 (“strongly disagree”) to 4 (“strongly agree”). Items 1, 6, 12, 17, and 19 were positive statements that were reverse-scored. The total score ranged from 0 to 96. The DS-MV uses the same cutoff (>30) as the original version for high demoralization. In this study, Cronbach’s alpha for the total scale was 0.96.

#### 2.3.2 Family resilience

Family resilience was assessed using the 32-item shortened Chinese version of the Family Resilience Assessment Scale (FRAS-C), which was developed by Sixbey (2005) and subsequently translated into Chinese by Li et al. (2016). It is a self–report scale, and each item is scored on a four-point Likert scale (1 = strongly disagree, 2 = disagree, 3 = agree, and 4 = strongly agree). The scale has three subscales: Family Communication and Problem Solving (FCPS), Utilizing Social Resources (USR), and Maintaining a Positive Outlook (MPO). The total score ranges from 32 to 128, with higher scores indicating greater family resilience. The scale demonstrated good reliability and validity. In this study, Cronbach’s alpha for the total scale was 0.99.

#### 2.3.3 Psychological resilience

Psychological resilience was measured by the Chinese version of the Conner and Davidson resilience scale (CD-RISC) (Connor and Davidson, 2003). The scale contains 13 questions on the resilience subscale, 8 questions on the strength subscale, and 4 questions on the optimism subscale, for a total of 25 questions. Each question is scored 0–4, with higher scores indicating better psychological resilience. A total score ≤25 indicates extremely poor psychological resilience, 26–50 indicates poor, 51–75 indicates average, and 76–100 indicates good. In this study, Cronbach’s alpha for the total scale was 0.92.

#### 2.3.4 Social support

The Perceived Social Support Scale (PSSS) was used to measure the level of social support of patients based on the original scale developed by Zimet et al. (1990). The scale contains four questions on the family subscale, four questions on the friend subscale, and four questions on the other subscales, for a total of 12 questions. Each topic was scored 1–7, with 12–36 indicating a low support level, 37–60 indicating a medium support level, and 61 or more indicating a high support level. In this study, the Cronbach’s alpha for the total scale was 0.95.

#### 2.3.5 Demographic and clinical characteristics

Demographic and clinical characteristics included gender, age, education, employment status, children, religion, marital status, cohabitation status, residence, monthly household income per capita, healthcare payment method, disease awareness, self-care ability, and time since diagnosis. These observational variables might influence DS. Hence, we considered these potential confounders and explored their effects on DS.

### 2.4 Data analysis

We conducted normality and homogeneity of variance tests. Normally distributed data are presented as mean ± standard deviation (SD), whereas skewed data are expressed as interquartile range (IQR). Statistical analyses were performed using SPSS 27.0, including descriptive statistics, nonparametric tests, Spearman’s correlation, and multivariate linear regression models. Structural equation modeling was used to examine the path relationships among the main study variables using IBM SPSS AMOS 28.0. The maximum likelihood method was used several times to fit the models. Using the Bootstrap method, resample the original data 5,000 times and conduct a mediation effect test with the 95% confidence interval (CI). The model fit indices included χ^2^/df, root mean square error of approximation (RMSEA), goodness of fit index (GFI), Tucker–Lewis index (TLI), incremental fit index (IFI), comparative fit index (CFI), and normed fit index (NFI). All statistical tests were bilateral, and statistical significance was set at *p* < 0.05.

### 2.5 Ethical considerations

This study was approved by the Ethics Committee of the Fourth People’s Hospital of Jinan (Approval No. LL20240054). Before the investigation began, the purpose and significance of the study were explained to decompensated patients with cirrhosis. Patients willing to participate in the study provided informed consent. The data were confidently held. If a participant dropped out, we respected their wishes.

## 3 Result

### 3.1 Sociodemographic characteristics

Table 1 shows the sociodemographic characteristics and DS status of the participants. A total of 260 patients with decompensated liver cirrhosis completed the study. More than half (61.15%) of the patients were male, with the majority falling within the 60–74 years age group (43.08%). In terms of educational attainment, a significant proportion completed primary school (34.23%) or junior high school (36.54%). Univariate analysis revealed that patients with decompensated liver cirrhosis with different cohabitation status and residence levels had significant differences in DS (*p* < 0.05).

**Table 1:** Comparison of general conditions of decompensated cirrhotic patients.

Variable	*N* (%)	DS-MV score M (P25 ~ P75)	*Z*/*χ*^2^	*p*
Gender			−0.445	0.656
Male	159 (61.15%)	23 (8 ~ 28)		
Female	101 (38.85%)	20 (6 ~ 28)		
Age			4.479	0.214
18–44	19 (7.31%)	25 (11 ~ 29)		
45–59	108 (41.54%)	24 (10 ~ 28)		
60–74	112 (43.08%)	17 (6.25 ~ 27)		
≥75	21 (8.07%)	16 (4.5 ~ 28)		
Education			3.313	0.346
Elementary school and below	89 (34.23%)	20 (6.5 ~ 27)		
Junior high school	95 (36.54%)	25 (9 ~ 29)		
Senior high school/vocational school	50 (19.23%)	14.5 (7.75 ~ 26)		
Associate degree and above	26 (10.00%)	18.5 (5.75 ~ 29)		
Employment status			6.162	0.104
Employed	43 (16.54%)	24 (13 ~ 28)		
Retired	64 (24.62%)	12.5 (5 ~ 29.75)		
Not in the workforce	24 (9.23%)	26 (9.25 ~ 33.25)		
Farmer	129 (49.61%)	20 (7.5 ~ 27)		
Children			3.555	0.314
0	8 (3.08%)	26 (13 ~ 40.75)		
1	104 (40.00%)	24 (7.25 ~ 30)		
2	111 (42.69%)	21 (7 ~ 28)		
≥3	37 (14.23%)	15 (7 ~ 25.5)		
Religion			1.577	0.115
No	232 (89.23%)	20 (7 ~ 28)		
Yes	28 (10.77%)	25 (13 ~ 32)		
Marital status			1.561	0.119
Married or cohabiting	237 (91.15%)	22 (7 ~ 28)		
Single/divorced/widowed	23 (8.85%)	19 (15 ~ 36)		
Cohabitation status			−1.969	0.049
Living alone	12 (4.62%)	26.5 (17.5 ~ 34.25)		
Living with others	248 (95.38%)	20 (7 ~ 28)		
Residence			−2.127	0.033
Rural	127 (48.85%)	24 (9 ~ 29)		
City	133 (51.15%)	16 (6.5 ~ 27)		
Monthly household income per capita			2.755	0.431
<1,500	64 (24.62%)	23.5 (7.75 ~ 28.75)		
1,500 ~ 1999	35 (13.46%)	24 (10 ~ 28)		
2000 ~ 2,999	54 (20.77%)	19.5 (7.75 ~ 27)		
≥3,000	107 (41.15%)	19 (6 ~ 28)		
Healthcare payment method			2.005	0.735
Employee medical insurance	69 (26.54%)	19 (7 ~ 28)		
Resident medical Insurance	69 (26.54%)	24 (8.5 ~ 29)		
Self-pay	3 (1.15%)	3 (2 ~ 18.5)		
Subsistence allowance	7 (2.69%)	19 (11 ~ 28)		
Others	112 (43.08%)	22.5 (7 ~ 27)		
Disease awareness			1.712	0.087
Fully informed	195 (75.00%)	22 (6 ~ 28)		
Partially informed	65 (25.00%)	22 (11 ~ 29)		
Self-care ability			7.675	0.053
No dependency	223 (85.77%)	19 (7 ~ 27)		
Mild dependency	23 (8.85%)	27 (14 ~ 30)		
Moderate dependency	13 (5.00%)	31 (12.5 ~ 56.5)		
Severe dependency	1 (0.38%)	27		
Time since diagnosis			8.293	0.141
<0.5 years	30 (11.54%)	13.5 (5 ~ 27)		
0.5 ~ 1 years	24 (9.23%)	22.5 (5 ~ 28)		
1 ~ 5 years	81 (31.15%)	24 (11 ~ 32.5)		
6 ~ 10 years	46 (17.69%)	23 (8.5 ~ 28)		
11 ~ 20 years	49 (18.85%)	15 (5.5 ~ 24.5)		
>20 years	30 (11.54%)	19 (6.75 ~ 27.5)		

The normality test revealed that the DS scores were not normally distributed; therefore, the median and interquartile ranges were used. The median DS score in patients with decompensated liver cirrhosis was 22 points (IQR, 7–28), with 48 patients (18.46%) scoring >30 points. Among the subscales, the “Sense of Failure” dimension had the highest median score of 6 points (IQR 5–7). See Supplementary Table S1 for details.

Subgroup analyses confirmed that significant negative correlations persisted after adjusting for all covariates. Interaction effects were non-significant across all subgroups (*p* > 0.05), except for the interaction between psychological resilience and age (*p* = 0.002), indicating robust results. See Supplementary Tables S2–S4 for details.

### 3.2 Correlation analysis

Table 2 shows the results of the correlation analysis for DS, psychological resilience, social support, and family resilience in decompensated patients with cirrhosis. The results showed that family resilience, psychological resilience, and social support were negatively correlated with DS. The results revealed a strong negative correlation between psychological resilience and DS (r = −0.738, *p* < 0.01), indicating that patients with higher psychological resilience are associated with lower levels of DS.

**Table 2:** Results of correlation analysis.

Variable	Demoralization syndrome					
	Demoralization	Loss of meaning	Dysphoria	Helplessness	Sense of failure	Disheartenment
Psychological resilience	−0.738**	−0.695**	−0.658**	−0.651**	−0.643**	−0.679**
Optimism	−0.602**	−0.545**	−0.514**	−0.530**	−0.533**	−0.566**
Strength	−0.668**	−0.640**	−0.579**	−0.566**	−0.608**	−0.606**
Resilience	−0.681**	−0.641**	−0.628**	−0.618**	−0.576**	−0.629**
Family resilience	−0.668**	−0.610**	−0.600**	−0.624**	−0.573**	−0.644**
FCPS	−0.660**	−0.610**	−0.598**	−0.619**	−0.558**	−0.639**
USR	−0.548**	−0.488**	−0.468**	−0.503**	−0.506**	−0.530**
MPO	−0.671**	−0.607**	−0.616**	−0.619**	−0.565**	−0.654**
Social support	−0.405**	−0.329**	−0.371**	−0.396**	−0.342**	−0.408**
Family support	−0.413**	−0.393**	−0.403**	−0.391**	−0.308**	−0.394**
Friend support	−0.326**	−0.243**	−0.303**	−0.313**	−0.288**	−0.325**
Other support	−0.352**	−0.272**	−0.342**	−0.371**	−0.295**	−0.354**

**p < 0.01.

### 3.3 Regression analysis

Table 3 shows the results of the multiple linear regression analysis. Collinearity diagnostics were performed on variables identified as statistically significant in the univariate and correlation analyses. The results showed that all variance inflation factors (VIF) were <5, indicating no multicollinearity among the independent variables. Multiple linear regression analysis revealed that family resilience, psychological resilience, and social support were significant factors influencing DS (*p* < 0.05). Cohabitation status and residence were not statistically significant in the linear regression analysis (*p*>0.05). This model explained 67.1% of the variance in DS among patients with decompensated liver cirrhosis.

**Table 3:** Results of multiple linear regression analysis.

Variable	*B*	SE	*β*	*t*	*p*	Tolerance	VIF	sr
Constant	99.286	5.817	–	17.068	<0.001	–	–	–
Cohabitation status	−1.157	2.601	−0.016	−0.445	0.657	0.930	1.075	−0.016
Residence	0.747	1.085	0.025	0.688	0.492	0.941	1.063	0.025
Psychological resilience	−0.562	0.058	−0.477	−9.707	<0.001	0.525	1.905	−0.346
Family resilience	−0.297	0.042	−0.364	−6.994	<0.001	0.468	2.135	−0.249
Social support	−0.137	0.053	−0.108	−2.580	0.010	0.729	1.373	−0.092

*R*^2^ = 0.678, adjusted *R*^2^ = 0.671, F = 106.796, VIF<5, no significant covariance in the independent variables and normally distributed residuals.

### 3.4 Mediation model

**Figure 1:**
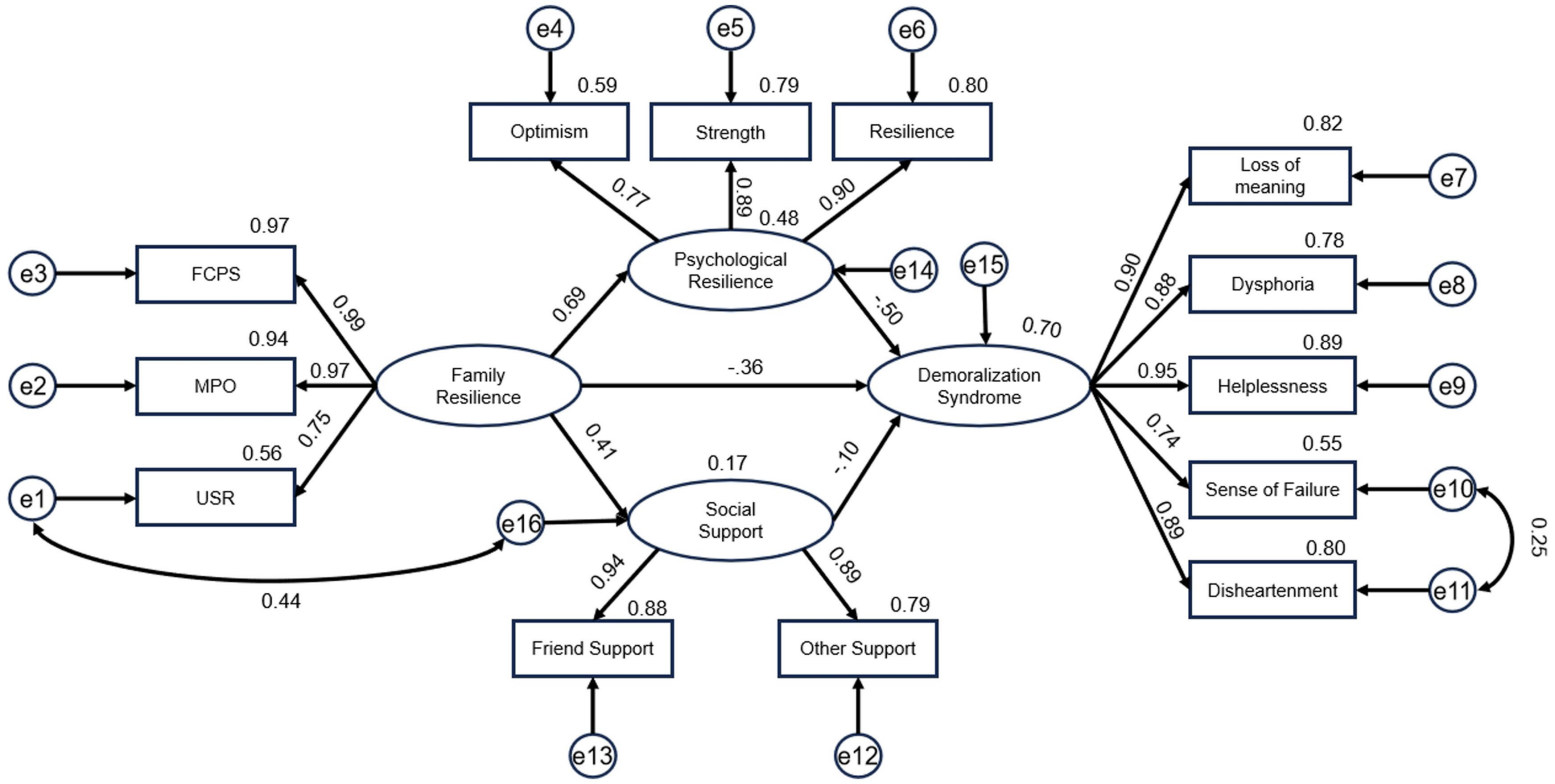
The mediating effect of psychological resilience and social support in the relationship between family resilience and demoralization syndrome in decompensated cirrhotic patients (Standard coefficients).

Figure 1 illustrates the path from family resilience to DS via psychological resilience and social support, with psychological resilience emerging as the most influential mediator, which aligns with Hypothesis. Table 4 shows that the five path coefficients were statistically significant (*p* < 0.05). Family resilience demonstrated a positive direct effect on psychological resilience (*β* = 0.695, *p* < 0.001) and a positive effect on social support (*β* = 0.406, *p* < 0.001). Conversely, psychological resilience exhibited a significant negative direct effect on DS (*β* = −0.500, *p* < 0.001). Family resilience also directly reduced DS (*β* = −0.360, *p* < 0.001), while social support showed a weaker but significant negative effect on DS (*β* = −0.104, *p* = 0.019). Both the utilizing social resources dimension of family resilience and social support involve accessing external assistance, but the former focuses on the family internal capacity to mobilize resources, while the latter emphasizes the actual acquisition of external support. The covariance between their error terms may reflect unmeasured common factors, such as willingness to seek resources. Meanwhile, the model was improved based on modification indices (Lin et al., 2025). Based on this, the structural equation model included a covariance between error terms e1 (pertaining to the utilizing social resources subdimension of family resilience) and e16 (pertaining to the latent variable social support). The model fit indices improved. Table 5 shows that the final model indicated a better fit (*χ*^2^/df = 2.578, RMSEA = 0.078, GFI = 0.925, NFI = 0.958). Based on the implied correlation matrix of latent variables from Amos output, multicollinearity diagnostics indicate that all inter-construct correlations are below the critical threshold of 0.85. See Supplementary Table S5 for details.

**Table 4:** Path analysis of structural equation model.

Path	Standardized coefficients	Unstandardized coefficients	SE	CR	*p*
Family resilience → Psychological resilience	0.695	1.064	0.111	9.578	***
Family resilience → Social support	0.406	1.328	0.206	6.453	***
Psychological resilience → Demoralization syndrome	−0.500	−0.598	0.080	−7.434	***
Family resilience → Demoralization syndrome	−0.360	−0.660	0.117	−5.629	***
Social support → Demoralization syndrome	−0.104	−0.058	0.025	−2.349	0.019

***p<0.001.

**Table 5:** Model fit of structural equation model.

Items	PCMIN/DF	RMSEA	GFI	NFI	TLI	CFI
Reference value	<3.0	<0.08	>0.9	>0.9	>0.9	>0.9
Removing e1-e16 covariance	3.282	0.094	0.908	0.945	0.948	0.961
Including e1-e16 covariance	2.578	0.078	0.925	0.958	0.964	0.973

Table 6 summarizes the direct, indirect, and total estimates of the model paths. Family resilience had a significant direct effect (*B* = −0.660, *p* < 0.001), accounting for 48.07% of the total effect. The indirect effects were mediated primarily through psychological resilience (*B* = −0.636, *p* < 0.001) and marginally through social support (*B* = −0.077, *p* = 0.010). The total indirect effect (*B* = −0.713, *p* < 0.001) explained 51.93% of the total effect. The results showed psychological resilience explained 46.32% of the variance and social support explained 5.61% of the variance.

**Table 6:** Total, direct, and indirect effect of family resilience on demoralization syndrome via psychological resilience and social support.

Items	Coeff/Effect	Proportion	*p*	95%CI	
				Lower	Upper
Direct effect	−0.660	48.07%	<0.001	−0.995	−0.356
Indirect effect					
*X* → M1 → *Y*	−0.636	46.32%	<0.001	−0.923	−0.434
*X* → M2 → *Y*	−0.077	5.61%	0.010	−0.154	−0.020
Total indirect effect	−0.713	51.93%	<0.001	−0.999	−0.499
Total effect	−1.373	100%	<0.001	−1.734	−1.103

*X*, family resilience; M1, psychological resilience; M2, social support; *Y,* demoralization syndrome.

## 4 Discussion

This study found that patients with decompensated cirrhosis exhibited a low level of DS, with a median total score of 22. However, Li et al. (2024) found the DS-MV score of inpatients with liver cirrhosis was (37.92 ± 12.85). This study found 48 patients (18.46%) demonstrated high levels of demoralization, representing a lower proportion of severe DS compared to the findings of other studies (Li et al., 2024; Hsu et al., 2022; Lee et al., 2012), which may be attributed to several factors. The survey found that patients with decompensated liver cirrhosis in this study demonstrated high family resilience (median = 97), moderate psychological resilience (median = 69), and high levels of social support (median = 64), which may be associated with the low prevalence of severe DS. Meanwhile, decompensated cirrhosis is often a chronic condition that allows patients to accept their reality gradually and prioritize their present quality of life, reducing their anxiety about the future. The questionnaires were administered during periods of clinical stability when the disease had minimal impact on patients’ psychological state. Additionally, the assessment scale for DS may be influenced by subjective self-reporting, as some patients may conceal their negative emotions during the evaluation.

This study is the first to investigate the association between family resilience and DS in patients with decompensated cirrhosis. Family resilience had a significant direct effect (95% CI: −0.995 to −0.356), accounting for 48.07% of the total effect. This study found that family resilience negatively predicted DS, which is consistent with the findings of Yan in patients with cleft lip and palate (Yan et al., 2023). Family resilience is the ability of a family to overcome multiple environmental stressors and rebound positively in the face of a major stressful event. Cirrhosis is a disease that has a significant impact on family systems. Companionship and support of family members help the patients adapt to the symptoms of cirrhosis and the suffering caused by complications, which helps reduce the feeling of helplessness and loneliness and, thus, reduces the level of DS.

This study found a significant parallel mediation effect of psychological resilience and social support (95% CI: −0.999 to −0.499), accounting for 51.93% of the total effect.

These findings suggested that psychological resilience played a significant mediating role between family resilience and DS—family resilience can reduce the risk of DS in patients with decompensated liver cirrhosis by enhancing psychological resilience. Psychological resilience explained 46.32% of the variance. This study found that family resilience significantly predicted psychological resilience, which aligns with the conclusions drawn by Qiu et al. (2021), who investigated the predictive role of family resilience on psychological resilience among Chinese patients undergoing maintenance hemodialysis. Maintaining a positive outlook within family resilience enables patients to confront difficulties with an optimistic attitude, effectively adapt to the suffering caused by liver cirrhosis, and recover, which may promote the enhancement of individual psychological resilience. This study revealed that psychological resilience could negatively predict DS, which is in general agreement with the findings of Zhang et al. (2025). Patients with high psychological resilience may demonstrate enhanced emotional regulation and problem-solving capacities, mitigating helplessness associated with DS. These findings suggest potential intervention strategies that focus on resilience enhancement to reduce demoralization among patients. In summary, family resilience may help patients with decompensated cirrhosis maintain positive cognitive abilities in adversity by cultivating psychological resilience as a key psychological resource, effectively preventing DS. This finding indicates that clinical healthcare professionals should prioritize enhancing patients’ psychological resilience when formulating strategies to reduce DS. This pathway provides an important theoretical basis for family interventions and mental-health promotion.

The results showed that social support played a significant mediating role between family resilience and DS in patients with decompensated liver cirrhosis, accounting for 5.61% of the total effect. A previous study indicated that patients who perceived lower social support might demonstrate higher family resilience (Cui et al., 2023), whereas in this study, family resilience could positively influence perceived social support. Therefore, further research is required to explore the relationship between perceived social support and family resilience. This study showed that social support for liver cirrhosis exerted a negative predictive effect on demoralization, which is in general agreement with Kang et al. (2023), who concluded that social support of cancer patients was a risk factor for demoralization. Wang C. et al. (2024) found that perceived social support was an important factor associated with the severity of demoralization in patients with burns. Social support can provide patients with multidimensional positive experiences at the physiological, psychological, and spiritual levels, which are conducive to the treatment of diseases. Patients with liver cirrhosis experience varying degrees of disease-related stigma, fear that others may discover their condition, feel shame and self-blame for the diagnosis, and occasionally face social exclusion or isolation. People with liver cirrhosis are more likely to avoid seeking help from others because of social stigma (Schomerus et al., 2022), which inevitably increases loneliness, isolation, and DS. In summary, family resilience may help prevent DS in patients with decompensated liver cirrhosis through a social support system. This study provides an important theoretical basis for family interventions and the development of social support initiatives targeting this patient population.

We need to consider the influence of cultural background on DS, particularly within Chinese society. For instance, the common multi-generational cohabitation structure in China may provide stronger family support, yet simultaneously heighten patient stress due to intergenerational value conflicts. Chinese cultural norms often involve implicit expectations regarding social support, emphasizing that “family scandals should not be publicized.” This may lead patients to resist seeking external help (Jin and Li, 2023)—including from professionals—delaying crucial assistance. Additionally, China’s collectivist values may cause patients to perceive their illness as a burden to the whole family, or as an inability to fulfill the duty of “bringing honor to the family.” These pressures can intensify feelings of hopelessness and frustration, thereby exacerbating DS.

Building on the relationship between family resilience, psychological resilience, social support and DS identified in this study, we propose a tripartite intervention framework to address decompensated cirrhosis patients’ DS: implementation of family-based psychoeducation programs, such as group sessions targeting demoralization reduction and family camping activities; provision of individual therapeutic interventions, including mindfulness-integrated cognitive behavioral therapy (Soleymani Moghadam et al., 2024), dignity therapy (Iani et al., 2020), and cognitive behavioral therapy (Gostoli et al., 2024); and establishing structured social support systems for cirrhosis patients through hospital-community collaboration, while conducting public education to disseminate cirrhosis knowledge and reduce societal prejudice.

Our study described the current status of DS in patients with decompensated liver cirrhosis in Jinan, Shandong Province, China, and examined the parallel mediating effects of psychological resilience and social support on the relationship between family resilience and DS.

### 4.1 Limitation

Although our study provides valuable insights into the effects of family resilience on DS, some limitations should be considered when interpreting the current results. First, the cross-sectional design could not establish causal relationships, and potential changes in DS over time could not be observed. Second, this study employed a self-report questionnaire, which may have contributed to reporting bias. Third, this study used convenience sampling and included a small sample size limited to Jinan in Shandong Province, future studies could employ stratified sampling to enhance external validity.

## 5 Conclusion

These findings provide a theoretical foundation for targeted interventions to reduce demoralization in decompensated patients with cirrhosis. Effective strategies should integrate family resilience, psychological resilience, and social support.

## Data Availability

The original contributions presented in the study are included in the article/Supplementary material, further inquiries can be directed to the corresponding author.
