# Supplementary material for: Family resilience and demoralization in decompensated cirrhosis: parallel mediation of psychological resilience and social support
Source: Front Psychol. 2025 Aug 1;16:1623122. doi: 10.3389/fpsyg.2025.1623122 (PMC12355604; doi:10.3389/fpsyg.2025.1623122)
Supplement: Supplementary file 1 [file Table_1.DOCX]

**Supplementary Table S1** The levels of demoralization syndrome, family resilience, psychological resilience and social support

| **Variable** | **Score** | **Item Count** |
| --- | --- | --- |
| **Demoralization syndrome** | 22(7～28) | 24 |
| Disheartenment | 5(1～7) | 6 |
| Loss of meaning | 3(0～5) | 5 |
| Dysphoria | 4(0～6) | 5 |
| Helplessness | 3(0～4) | 4 |
| Sense of failure | 6(5～7) | 4 |
| **Family resilience** | 97(95～124) | 32 |
| FCPS | 70(68～90) | 23 |
| USR | 9(9～11) | 3 |
| MPO | 18(18～24) | 6 |
| **Psychological resilience** | 69(61～75) | 25 |
| Optimism | 10(8～12) | 4 |
| Strength | 23(20～25) | 8 |
| Resilience | 35.5(32～39) | 13 |
| **Social support** | 64(56～72) | 12 |
| Family support | 24(24～26) | 4 |
| Friend support | 20(16～24) | 4 |
| Other support | 20(16～24) | 4 |
