# Supplementary material for: Family resilience and demoralization in decompensated cirrhosis: parallel mediation of psychological resilience and social support
Source: Front Psychol. 2025 Aug 1;16:1623122. doi: 10.3389/fpsyg.2025.1623122 (PMC12355604; doi:10.3389/fpsyg.2025.1623122)
Supplement: Supplementary file 2 [file Table_2.DOCX]

**Supplementary Table S2** Subgroup analyses of the association between family resilience and demoralization syndrome

| **Subgroup** | **n** | **crude.Coefficient 95CI** | **crude.P value** | **adj.Coefficient 95CI** | **adj.P value** | **P for interaction** |
| --- | --- | --- | --- | --- | --- | --- |
| **Gender** |  |  |  |  |  |  |
| Male | 159 | -0.57 (-0.66~-0.48) | <0.001 | -0.53 (-0.62~-0.44) | <0.001 | 0.272 |
| Female | 101 | -0.64 (-0.74~-0.53) | <0.001 | -0.62 (-0.75~-0.5) | <0.001 |  |
| **Age** |  |  |  |  |  |  |
| 18-44 | 19 | -0.51 (-0.72~-0.3) | <0.001 | -0.48 (-0.87~-0.09) | 0.073 | 0.621 |
| 45-59 | 108 | -0.6 (-0.72~-0.48) | <0.001 | -0.59 (-0.72~-0.46) | <0.001 |  |
| 60-74 | 112 | -0.6 (-0.7~-0.49) | <0.001 | -0.59 (-0.71~-0.47) | <0.001 |  |
| ≥75 | 21 | -0.65 (-0.82~-0.48) | <0.001 | -0.87 (-1.15~-0.6) | 0.001 |  |
| **Cohabitation status** |  |  |  |  |  |  |
| Living alone | 12 | -0.68 (-0.91~-0.44) | <0.001 | -0.55 (NaN~NaN) | NaN | 0.935 |
| Living with others | 248 | -0.6 (-0.67~-0.53) | <0.001 | -0.57 (-0.65~-0.5) | <0.001 |  |
| **Residence** |  |  |  |  |  |  |
| Rural | 127 | -0.58 (-0.67~-0.48) | <0.001 | -0.56 (-0.67~-0.45) | <0.001 | 0.599 |
| City | 133 | -0.62 (-0.71~-0.52) | <0.001 | -0.56 (-0.65~-0.46) | <0.001 |  |
| **Time since diagnosis** |  |  |  |  |  |  |
| <0.5 years | 30 | -0.65 (-0.82~-0.48) | <0.001 | -0.56 (-0.79~-0.33) | <0.001 | 0.361 |
| 0.5~1 years | 24 | -0.47 (-0.65~-0.29) | <0.001 | -0.23 (-0.56~0.1) | 0.211 |  |
| 1~5 years | 81 | -0.55 (-0.69~-0.41) | <0.001 | -0.53 (-0.67~-0.4) | <0.001 |  |
| 6~10 years | 46 | -0.73 (-0.93~-0.52) | <0.001 | -0.8 (-1.02~-0.58) | <0.001 |  |
| 11~20 years | 49 | -0.58 (-0.73~-0.43) | <0.001 | -0.6 (-0.81~-0.38) | <0.001 |  |
| >20 years | 30 | -0.68 (-0.82~-0.54) | <0.001 | -0.55 (-0.81~-0.28) | 0.001 |  |
| **Monthly household income per capita** |  |  |  |  |  |  |
| ＜1500 | 64 | -0.67 (-0.8~-0.54) | <0.001 | -0.62 (-0.8~-0.45) | <0.001 | 0.468 |
| 1500~1999 | 35 | -0.58 (-0.8~-0.36) | <0.001 | -0.51 (-0.69~-0.32) | <0.001 |  |
| 2000~2999 | 54 | -0.5 (-0.64~-0.35) | <0.001 | -0.56 (-0.77~-0.35) | <0.001 |  |
| ≥3000 | 107 | -0.6 (-0.71~-0.49) | <0.001 | -0.54 (-0.65~-0.44) | <0.001 |  |

Note: Adjusted for gender, age, cohabitation status, residence, time since diagnosis, and monthly household income per capita.
