# Supplementary material for: Family resilience and demoralization in decompensated cirrhosis: parallel mediation of psychological resilience and social support
Source: Front Psychol. 2025 Aug 1;16:1623122. doi: 10.3389/fpsyg.2025.1623122 (PMC12355604; doi:10.3389/fpsyg.2025.1623122)
Supplement: Supplementary file 3 [file Table_3.DOCX]

**Supplementary Table S3** Subgroup analyses of the association between psychological resilience and demoralization syndrome

| **Subgroup** | **n** | **crude.Coefficient 95CI** | **crude.P value** | **adj.Coefficient 95CI** | **adj.P value** | **P for interaction** |
| --- | --- | --- | --- | --- | --- | --- |
| **Gender** |  |  |  |  |  |  |
| Male | 159 | -0.84 (-0.96~-0.72) | <0.001 | -0.82 (-0.94~-0.69) | <0.001 | 0.379 |
| Female | 101 | -0.99 (-1.14~-0.83) | <0.001 | -1 (-1.18~-0.81) | <0.001 |  |
| **Age** |  |  |  |  |  |  |
| 18-44 | 19 | -0.65 (-0.82~-0.49) | <0.001 | -0.54 (-0.86~-0.22) | 0.03 | 0.002 |
| 45-59 | 108 | -0.83 (-0.99~-0.68) | <0.001 | -0.81 (-0.99~-0.64) | <0.001 |  |
| 60-74 | 112 | -0.95 (-1.1~-0.81) | <0.001 | -0.95 (-1.11~-0.79) | <0.001 |  |
| ≥75 | 21 | -1.5 (-1.85~-1.16) | <0.001 | -1.38 (-2.01~-0.74) | 0.006 |  |
| **Cohabitation status** |  |  |  |  |  |  |
| Living alone | 12 | -0.92 (-1.31~-0.52) | 0.001 | -0.08 (NaN~NaN) | NaN | 0.919 |
| Living with others | 248 | -0.88 (-0.98~-0.79) | <0.001 | -0.88 (-0.98~-0.78) | <0.001 |  |
| **Residence** |  |  |  |  |  |  |
| Rural | 127 | -0.89 (-1.02~-0.77) | <0.001 | -0.89 (-1.03~-0.76) | <0.001 | 0.796 |
| City | 133 | -0.91 (-1.05~-0.76) | <0.001 | -0.83 (-0.99~-0.68) | <0.001 |  |
| **Time since diagnosis** |  |  |  |  |  |  |
| <0.5 years | 30 | -0.91 (-1.19~-0.62) | <0.001 | -0.83 (-1.21~-0.45) | 0.001 | 0.611 |
| 0.5~1 years | 24 | -1.07 (-1.5~-0.64) | <0.001 | -0.88 (-1.49~-0.26) | 0.021 |  |
| 1~5 years | 81 | -0.79 (-0.95~-0.63) | <0.001 | -0.76 (-0.94~-0.58) | <0.001 |  |
| 6~10 years | 46 | -0.94 (-1.17~-0.72) | <0.001 | -1.04 (-1.27~-0.8) | <0.001 |  |
| 11~20 years | 49 | -0.91 (-1.16~-0.66) | <0.001 | -1.05 (-1.36~-0.74) | <0.001 |  |
| >20 years | 30 | -0.96 (-1.19~-0.74) | <0.001 | -0.65 (-0.92~-0.37) | <0.001 |  |
| **Monthly household income per capita** |  |  |  |  |  |  |
| ＜1500 | 64 | -0.98 (-1.16~-0.81) | <0.001 | -0.93 (-1.12~-0.73) | <0.001 | 0.447 |
| 1500~1999 | 35 | -0.7 (-0.93~-0.47) | <0.001 | -0.67 (-0.98~-0.35) | <0.001 |  |
| 2000~2999 | 54 | -0.66 (-0.89~-0.44) | <0.001 | -0.68 (-0.92~-0.43) | <0.001 |  |
| ≥3000 | 107 | -1.03 (-1.19~-0.88) | <0.001 | -0.98 (-1.14~-0.82) | <0.001 |  |

Note: Adjusted for gender, age, cohabitation status, residence, time since diagnosis, and monthly household income per capita.
