# Supplementary material for: Family resilience and demoralization in decompensated cirrhosis: parallel mediation of psychological resilience and social support
Source: Front Psychol. 2025 Aug 1;16:1623122. doi: 10.3389/fpsyg.2025.1623122 (PMC12355604; doi:10.3389/fpsyg.2025.1623122)
Supplement: Supplementary file 4 [file Table_4.DOCX]

**Supplementary Table S4** Subgroup analyses of the association between social support and demoralization syndrome

| **Subgroup** | **n** | **crude.Coefficient 95CI** | **crude.P value** | **adj.Coefficient 95CI** | **adj.P value** | **P for interaction** |
| --- | --- | --- | --- | --- | --- | --- |
| **Gender** |  |  |  |  |  |  |
| Male | 159 | -0.57 (-0.74~-0.4) | <0.001 | -0.52 (-0.69~-0.35) | <0.001 | 0.476 |
| Female | 101 | -0.69 (-0.92~-0.46) | <0.001 | -0.65 (-0.89~-0.42) | <0.001 |  |
| **Age** |  |  |  |  |  |  |
| 18-44 | 19 | -0.39 (-0.74~-0.04) | 0.041 | -0.27 (-1.41~0.86) | 0.66 | 0.075 |
| 45-59 | 108 | -0.54 (-0.75~-0.32) | <0.001 | -0.55 (-0.78~-0.32) | <0.001 |  |
| 60-74 | 112 | -0.69 (-0.9~-0.48) | <0.001 | -0.71 (-0.95~-0.47) | <0.001 |  |
| ≥75 | 21 | -1.28 (-1.89~-0.67) | 0.001 | -1.26 (-2.66~0.14) | 0.128 |  |
| **Cohabitation status** |  |  |  |  |  |  |
| Living alone | 12 | -0.59 (-1.05~-0.14) | 0.027 | -0.2 (NaN~NaN) | NaN | 0.332 |
| Living with others | 248 | -0.61 (-0.75~-0.46) | <0.001 | -0.6 (-0.74~-0.45) | <0.001 |  |
| **Residence** |  |  |  |  |  |  |
| Rural | 127 | -0.64 (-0.83~-0.45) | <0.001 | -0.65 (-0.85~-0.45) | <0.001 | 0.531 |
| City | 133 | -0.58 (-0.78~-0.39) | <0.001 | -0.54 (-0.73~-0.35) | <0.001 |  |
| **Time since diagnosis** |  |  |  |  |  |  |
| <0.5 years | 30 | -0.41 (-0.78~-0.03) | 0.044 | -0.66 (-1.01~-0.3) | 0.003 | 0.63 |
| 0.5~1 years | 24 | -0.64 (-1.1~-0.18) | 0.012 | -0.77 (-1.36~-0.18) | 0.031 |  |
| 1~5 years | 81 | -0.59 (-0.84~-0.33) | <0.001 | -0.59 (-0.85~-0.32) | <0.001 |  |
| 6~10 years | 46 | -0.71 (-1.15~-0.27) | 0.003 | -0.85 (-1.38~-0.31) | 0.004 |  |
| 11~20 years | 49 | -0.57 (-0.88~-0.26) | 0.001 | -0.62 (-1.03~-0.2) | 0.006 |  |
| >20 years | 30 | -0.84 (-1.12~-0.55) | <0.001 | -0.6 (-0.94~-0.26) | 0.003 |  |
| **Monthly household income per capita** |  |  |  |  |  |  |
| ＜1500 | 64 | -0.73 (-1.01~-0.45) | <0.001 | -0.77 (-1.04~-0.49) | <0.001 | 0.958 |
| 1500~1999 | 35 | -0.32 (-0.67~0.03) | 0.087 | -0.26 (-0.59~0.07) | 0.135 |  |
| 2000~2999 | 54 | -0.73 (-0.99~-0.46) | <0.001 | -0.78 (-1.08~-0.48) | <0.001 |  |
| ≥3000 | 107 | -0.59 (-0.81~-0.36) | <0.001 | -0.49 (-0.72~-0.26) | <0.001 |  |

Note: Adjusted for gender, age, cohabitation status, residence, time since diagnosis, and monthly household income per capita.
