# Supplementary material for: Family resilience and demoralization in decompensated cirrhosis: parallel mediation of psychological resilience and social support
Source: Front Psychol. 2025 Aug 1;16:1623122. doi: 10.3389/fpsyg.2025.1623122 (PMC12355604; doi:10.3389/fpsyg.2025.1623122)
Supplement: Supplementary file 5 [file Table_5.DOCX]

**Supplementary Table S5** Latent variable correlation matrix

| **Latent variable** | **Family**  **resilience** | **Social**  **support** | **Psychological**  **resilience** | **Demoralization syndrome** |
| --- | --- | --- | --- | --- |
| **Family resilience** | 1.000 |  |  |  |
| **Social support** | 0.406 | 1.000 |  |  |
| **Psychological resilience** | 0.695 | 0.282 | 1.000 |  |
| **Demoralization syndrome** | -0.750 | -0.391 | -0.779 | 1.000 |
